# Evaluation of cefiderocol trailing and other unclear susceptibility testing endpoints for *Acinetobacter* spp.

**DOI:** 10.1128/jcm.00186-26

**Published:** 2026-08-20

**Authors:** Mariana Castanheira, Joshua M. Maher, John H. Kimbrough, Maura H. Karr, Rodrigo E. Mendes

**Affiliations:** 1 Element Iowa City/JMI Laboratories 138461, North Liberty, Iowa, USA; Children's Hospital Los Angeles, Los Angeles, California, USA

**Keywords:** trailling, *Acinetobacter*, cefiderocol

## Abstract

Broth microdilution (BMD) susceptibility testing with *Acinetobacter* spp. can be challenging with skipped wells and trailing observed for some agents. This also applies to cefiderocol, where isolates can display different growth patterns, such as trailing, regrowth, or hazy growth. To better understand the growth patterns observed with cefiderocol BMD testing, the content of wells was evaluated microscopically. Analysis of the trailing, hazy, and regrowth wells above the MIC endpoints showed disturbed growth with long filamentous and spherical cells characteristic of cell envelope instability observed with other β-lactams. When a cell metabolic activity reagent was used to evaluate the viability of the cells in wells above the MIC, inconclusive results were obtained, but when plated, substantial growth was noted for most wells, demonstrating that those cells were able to escape cefiderocol’s bactericidal activity. Replicate testing (*n* = 5) showed that aberrant growth patterns were reproducible, with some MIC variability observed with isolates showing regrowth or trailing, and did not correlate with growth patterns that can be noted in cefiderocol disk diffusion testing (inner colonies, ghost zones, or fuzzy edges). When the prevalence of the different BMD growth patterns was evaluated with 455 isolates from Europe and the United States tested as part of a surveillance program for cefiderocol, about 60% of the isolates exhibited unclear endpoints, and these were noted in most countries, *Acinetobacter* species, and different genetic backgrounds. Unclear endpoints are not uncommon when testing *Acinetobacter* spp. against cefiderocol, and tolerability of cells in the presence of cefiderocol seems to be the cause of this.

**IMPORTANCE:**

Cefiderocol susceptibility testing for *Acinetobacter* spp. is challenging due to the aberrant growth patterns that occur in the broth microdilution method, including trailing, regrowth, and haziness. We initiated this study to better understand these phenomena and assess if these growth patterns were reproducible. We concluded that cefiderocol is not bactericidal against certain isolates and that, despite the cell membrane biosynthesis process being impaired, the cells do not undergo lysis as expected with β-lactam agents. We also evaluated the prevalence of these aberrant growth patterns and evaluated the genetic traits of the isolates producing them, showing that these patterns are common and not related to geographic origin or specific genetic traits.

## INTRODUCTION

*Acinetobacter* species displaying high rates of carbapenem resistance are often multidrug resistant (MDR). Only limited therapeutic options are available for the treatment of infections caused by these organisms (1, 2). Among *Acinetobacter* species, *Acinetobacter baumannii* is the most common cause of human infections, and this species displays higher resistance levels when compared to other *Acinetobacter* species (3, 4). The CDC highlights carbapenem-resistant *A. baumannii* (CRAB) isolates as an urgent threat to human health. In 2017, 8,500 patients hospitalized in the United States had infections caused by CRAB isolates, resulting in 700 deaths (5). A study evaluating the outcomes of patients infected with MDR *Acinetobacter* compared to non-MDR isolates in two US hospitals demonstrated that inappropriate antimicrobial therapy was associated with longer hospital stays when compared to infections caused by non-MDR *Acinetobacter* isolates, despite mortality rates being similar among the two groups (6).

Susceptibility testing results are pivotal to determining appropriate antimicrobial treatment, including potential therapy escalation and de-escalation (7), but testing challenges with *Acinetobacter* spp. have been reported for several agents, including β-lactams and β-lactam/β-lactamase inhibitor combinations that ehxibit trailing (8–10). In a study establishing a correlation between Clinical and Laboratory Standards Institute (CLSI) reference broth microdilution (BMD) and disk diffusion methods against 186 *Acinetobacter* spp. isolates, Swenson et al. noticed high error rates with ampicillin-sulbactam, piperacillin, piperacillin-tazobactam, ceftazidime, and cefepime (8). The authors attribute these errors to the subtle growth patterns observed in the BMD method that are difficult to interpret, including trailing. Similar difficult-to-interpret endpoints were observed by Hawley et al. (11) when testing β-lactam agents against 142 *Acinetobacter baumannii-calcoaceticus* species complex from deployed US military personnel and by Higgins et al. when testing various β-lactams alone and combined with sulbactam, tazobactam, and clavulanate against 115 *A. baumannii* isolates (9).

Cefiderocol is a siderophore cephalosporin conjugate molecule that utilizes bacterial iron-transport systems for cell entry (10). Once inside the periplasm, the cephalosporin moiety inhibits penicillin-binding proteins and disrupts peptidoglycan biosynthesis (12). Cefiderocol exhibits activity against gram-negative isolates, multidrug-resistant organisms, and those displaying carbapenem resistance, including *Acinetobacter* species (13).

Cefiderocol BMD susceptibility testing needs to be performed in iron-depleted cation-adjusted Mueller-Hinton broth (IDCAMHB) as approved by the CLSI and European Committee on Antimicrobial Susceptibility Testing (EUCAST) organizations (14, 15) because MIC values obtained in that medium are highly predictive for efficacy in animal models (16, 17). However, this modification does not prevent the occurrence of complex growth patterns, including trailing, haziness, or regrowth, which complicate MIC endpoint determinations that have been noted in particular with *Acinetobacter* species (14–16).

In this study, we investigated the mechanism(s) underlying the trailing phenotypes observed with cefiderocol BMD susceptibility testing of *A. baumannii*, examined the prevalence of isolates with such unclear endpoints in a global surveillance program, and studied the epidemiological characteristics of those isolates. Additionally, we evaluated if those phenotypes affect disk diffusion testing.

## RESULTS

### Isolates and broth microdilution method results

**Fig 1:**
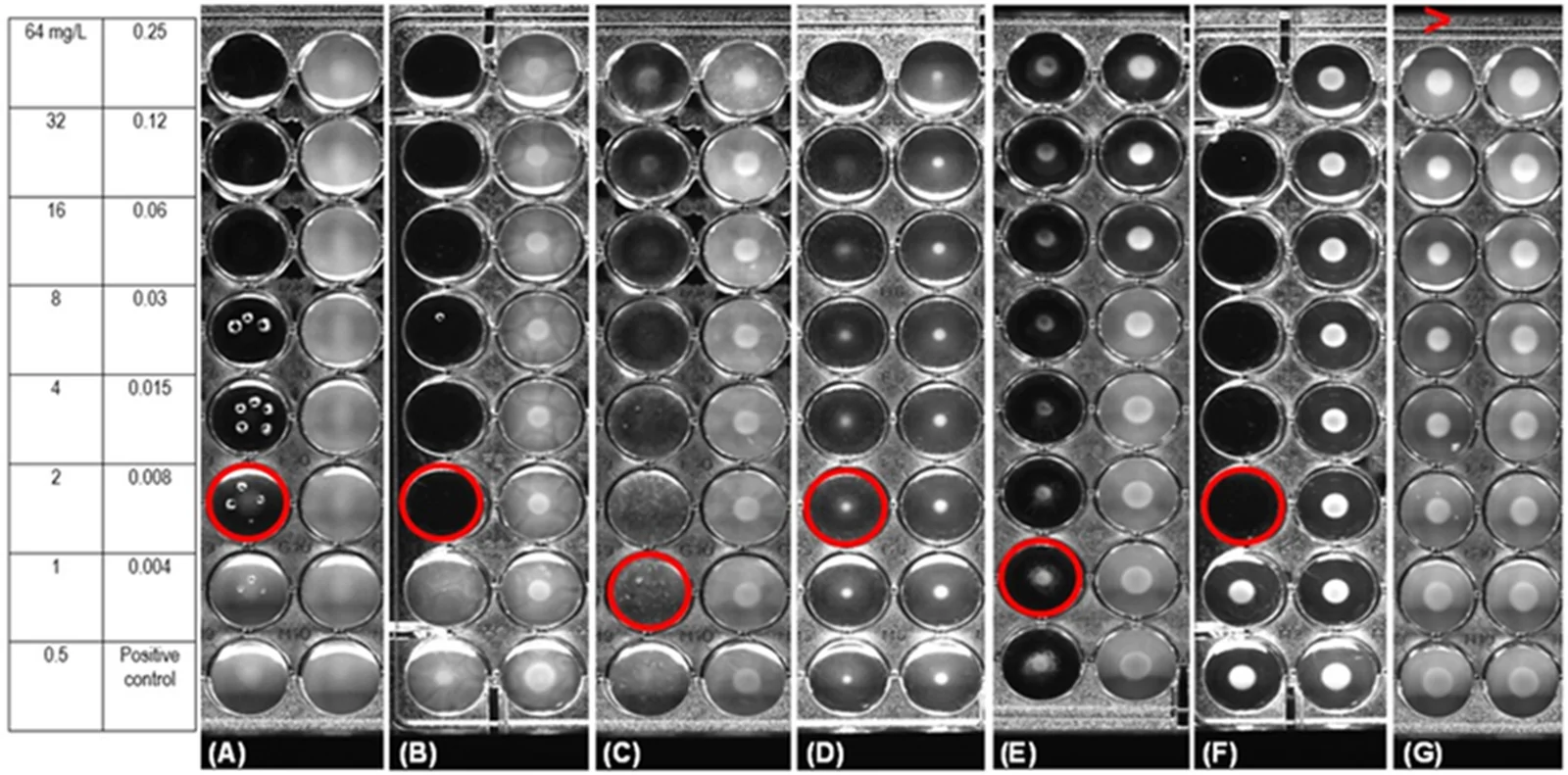
Broth microdilution endpoint growth patterns of *Acinetobacter* spp. tested against cefiderocol: hazy (**A**), hazy endpoint (**B**), regrowth (**C**), trailing (**D**), trailing all the way out (**E**), susceptible (**F**), and resistant (**G**). The testing concentrations are displayed on the left of the figure, and MIC values are circled in red.

Twenty-three clinical *Acinetobacter* spp. isolates displaying five distinct growth patterns when initially tested with the reference broth microdilution method for cefiderocol susceptibility testing in IDCAMHB were selected for this study (Table 1). The endpoint growth phenotypes were defined as trailing endpoint (reduced but persistent growth at concentrations above the MIC), hazy growth (homogenous suspension of turbid growth), regrowth reminiscent of the “Eagle effect” (18), and in-range resistant or susceptible with a clear growth endpoint (Fig. 1A, C, D, F, G). Each aberrant phenotype was represented by five isolates according to their initial susceptibility testing results, and four isolates, each with clear susceptible or resistant endpoints, were examined. Twenty-one isolates in this set belonged to the *A. baumannii-calcoaceticus* species complex, and one each of *Acinetobacter lwoffii* and *Acinetobacter radioresistens* were included.

**TABLE 1:** Cefiderocol susceptibility testing results and growth patterns (examples in Fig. 1) for 23 *Acinetobacter* spp. isolates^*c*^

Growth pattern	Organism^*a*^	Cefiderocol					
		Initial MIC (mg/L)	MIC (mg/L)	Modal MIC (mg/L)	MBC	Disk zone (30 µg, mm)	Disk zone diameter pattern (Fig. 2 panel if available)
Susceptible	ABC complex	0.03	0.03, 0.03, 0.03, 0.06, 0.06	0.03	0.06	24, 24, 24	Clear zone
Susceptible	ABC complex	0.06	0.03, 0.03, 0.03, 0.06, 0.12,	0.03	0.25	28, 29, 29	Clear zone^*b*^
Susceptible	ABC complex	0.06	0.03, 0.03, 0.03, 0.03, 0.06	0.03	0.06	29, 29, 30	Clear zone
Susceptible	ABC complex	0.5	0.12, 0.12, 0.25, 0.5, 0.5	0.5	16	22, 22, 23	Clear zone
Resistant	ABC complex	8	2, 2, 2, 2, 4	2	>64	6, 6, 6	Inner colonies (A1)
Resistant	ABC complex	16	2, 4, 4, 4, 4	4	64	6, 6, 6	Inner colonies (A2)
Resistant	ABC complex	>64	0.5, 1, 2, 2, 2	2	>64	22, 22, 23	Clear zone
Resistant	ABC complex	>64	>64, >64, >64, >64, >64	>64	>64	6, 6, 6	Inner colonies (A3)
Regrowth	ABC complex	0.25	0.12, 0.12, 0.12, 0.25, 0.5	0.12	>64	21, 22, 22	Clear zone
Regrowth	ABC complex	0.25	0.06, 0.06, 0.06, 0.06, 1	0.06	>64	23, 25, 25	Clear zone (B1)
Regrowth	ABC complex	0.25	0.25, 0.5, 0.5, 0.5, 1	0.5	>64	20, 20, 21	Few inner colonies in one replicate (B2)
Regrowth	ABC complex	2	1, 1, 1, 1, 2	1	>64	13, 16, 17	Inner colonies (B3)
Regrowth	*A. lwoffii*	0.5	0.5, 0.5, 0.5, 0.5, 1	0.5	1	24, 24, 25	Clear zone
Hazy	ABC complex	0.015	0.25, 0.5, 0.5, 1, 1	1	>64	25, 26, 27	Clear zone
Hazy	ABC complex	0.25	0.12, 0.25, 0.25, 0.5, 0.5	0.5	>64	25, 26, 26	Clear zone (C1)
Hazy	ABC complex	0.25	0.25, 0.25, 0.5, 0.5, 1	0.5	>64	25, 25, 26	Clear zone
Hazy	ABC complex	2	1, 1, 2, 2, 4	2	>64	26, 27, 27	Inner colonies (C2)
Hazy	ABC complex	4	1, 2, 2, 4, 4	2	>64	14, 15, 15	Clear zone (C3)
Trailing	ABC complex	0.25	0.12, 0.12, 0.12, 0.25, 0.25	0.12	32	22, 23, 23	Clear zone
Trailing	ABC complex	0.25	0.06, 0.12, 0.12, 0.25, 0.5	0.12	>64	20, 21, 21	Fuzzy edges (D1)
Trailing	ABC complex	1	0.5, 0.5, 0.5, 2, 2	0.5	>64	21, 22, 22	Ghost zone (D2)
Trailing	ABC complex	1	0.5, 1, 1, 1, 2	1	>64	6, 6, 6	Double halo and ghost zone (D3)
Trailing	*A. radioresistens*	0.5	0.03, 0.03, 0.03, 0.06, 0.06	0.03	>64	25, 28, 28	Clear zone

^*a*^ ABC complex, *Acinetobacter baumannii-calcoaceticus* complex.

^*b*^ One replicate had a few inner colonies.

^*c*^ Patterns were determined based on initial cefiderocol MIC testing.

The initial cefiderocol MIC values for the 23 *Acinetobacter* spp. isolates are listed in Table 1. Isolates were then retested in five replicates, each prepared from a different inoculum, using the same IDCAMHB and susceptibility panel lot and a single reader to determine endpoints for consistency. Growth patterns and MIC values were reproducible among the five replicates, and only 3 out of 23 isolates displayed cefiderocol MIC values with results between the lowest and highest of the five replicates that exceeded two doubling dilutions. Isolates displaying this reduced reproducibility exhibited trailing (two isolates) or regrowth (one isolate) phenotypes for which distinct MIC values are expectedly more challenging to determine. For 17 isolates, the modal MIC was within one doubling dilution from the initially determined MIC value. For the six isolates with more discrepant results, all except one isolate showed reduced modal MIC values compared to the initial MIC. For three isolates initially categorized as resistant (initial MICs of 8, 16, and >64 mg/L), the modal MICs of the replicates (2, 4, and 2 mg/L, respectively) fell within the CLSI susceptible range (≤4 mg/L), such that the categorical interpretation changed from resistant to susceptible; no isolate changed from susceptible to resistant.

### Disk diffusion

**Fig 2:**
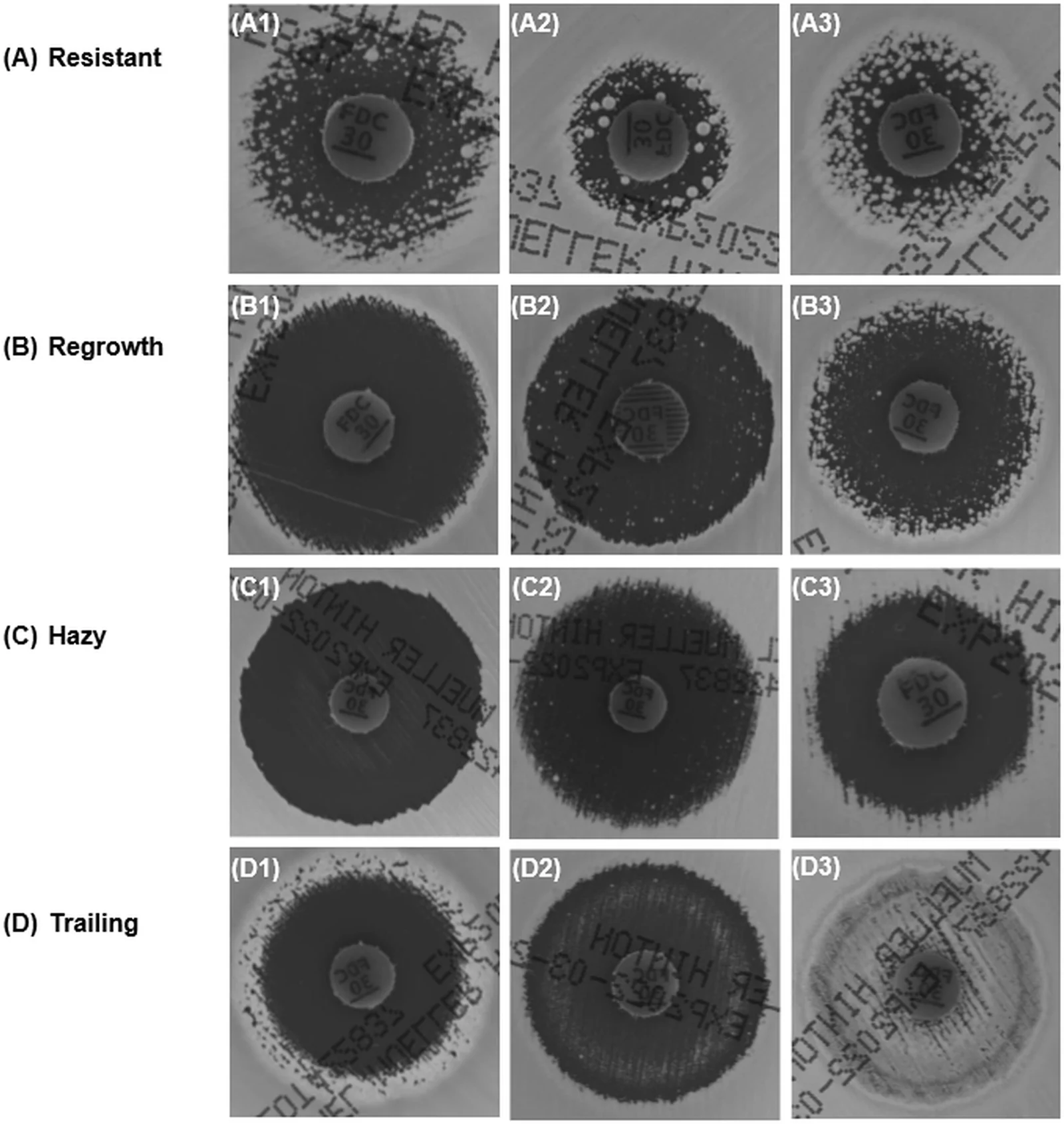
Cefiderocol disk zone examples for phenotypes noted with *Acinetobacter* spp. isolates.

The 23 *Acinetobacter* spp. isolates were tested in triplicate by the disk diffusion method on Mueller-Hinton agar (MHA) using a commercially available 30 μg cefiderocol disk. All four susceptible isolates displayed a clear zone of inhibition (Table 1), except for one replicate of one isolate that exhibited a few inner colonies within the zone of inhibition. For the four resistant isolates, one exhibited clear zones of inhibition, and the remaining isolates displayed inner colonies in the inhibition zone that, in two instances, went all the way to the disk (Fig. 2A1 through A3). Three regrowth and four hazy phenotype isolates displayed clear disk zones with or without fuzzy edges (Fig. 2B1 and C1), but one for each of these phenotypes had inner colonies in all three replicates (Fig. 2B2 and C2), and one regrowth isolate had inner colonies in one replicate (Fig. 2B3). Two trailing phenotype isolates displayed a clear zone of inhibition. The remaining trailing phenotype isolates had fuzzy edges (Fig. 2D1), ghost zones (Fig. 2D2), or a double halo and ghost zone with growth all the way to the disk (Fig. 2D3).

### Minimum bactericidal concentrations and cell viability

*Acinetobacter* spp. isolates were tested to determine the minimum bactericidal concentration (MBC), and 20 of the isolates produced cefiderocol MBC/MIC ratios >4, showing a lack of bactericidal activity against those isolates (Table 1). For 16 isolates, the cefiderocol MBC values were >64 mg/L and wells at or above the MIC produced a lawn of growth when plated on agar media. For only three isolates, the cefiderocol MBC/MIC ratios were ≤4, indicating bactericidal activity: two susceptible isolates and one displaying a regrowth phenotype.

**Fig 3:**
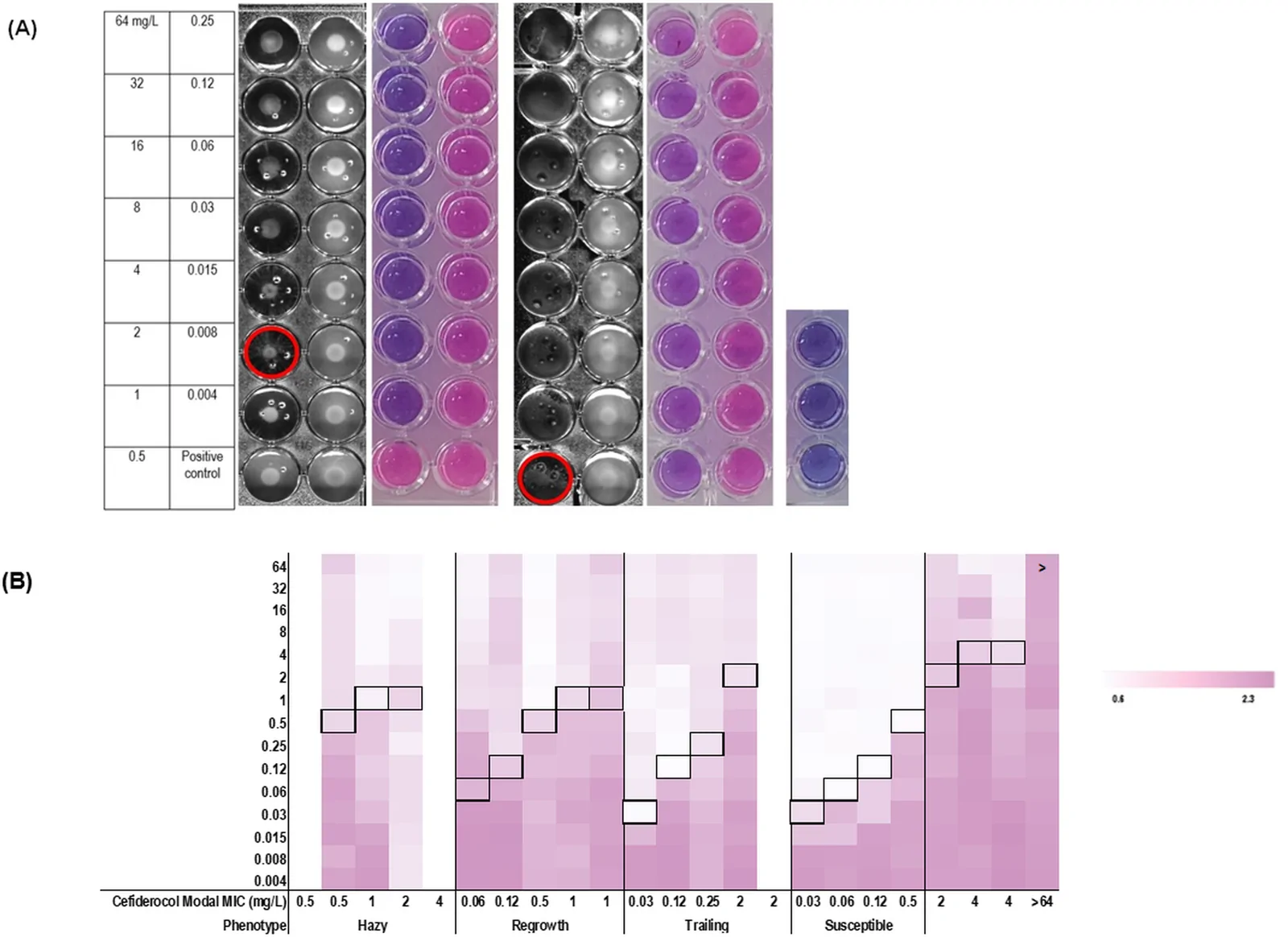
(**A**) Cefiderocol broth microdilution panels before and after incubation with alamarBlue. MIC values are circled in red; negative controls are on the right; and the cefiderocol concentrations tested are displayed on the left. (**B**) Optical density measurements (OD_570_) for cefiderocol susceptibility testing using a cell viability indicator. Higher absorbance, indicated by a darker pink color, correlates with increased cell viability. Visual MIC endpoint reads are outlined in black.

After visual endpoint determination, viability of the BMD cefiderocol panels was also assessed by the addition of resazurin, a colorimetric cell viability indicator that changes from blue to pink in the presence of metabolically active cells. Eleven (11/23) isolates had no visual color change at an optical density (OD) of 570 nm in wells above the cefiderocol MIC endpoint, indicating an absence of metabolically active cells (Fig. 3A). Expectedly, all four susceptible isolates had no metabolically active cells above the well where the MIC endpoint was determined, and eight isolates displaying turbidity above the MIC that was ignored also displayed no change in color (Fig. 3B). These isolates displayed regrowth (*n* = 1), trailing (*n* = 3), resistance (*n* = 1), and a hazy growth pattern (*n* = 2). Nine *Acinetobacter* spp. isolates had metabolically active cells in wells with concentrations of cefiderocol higher than the MIC endpoint.

### Microscopy observations

**Fig 4:**
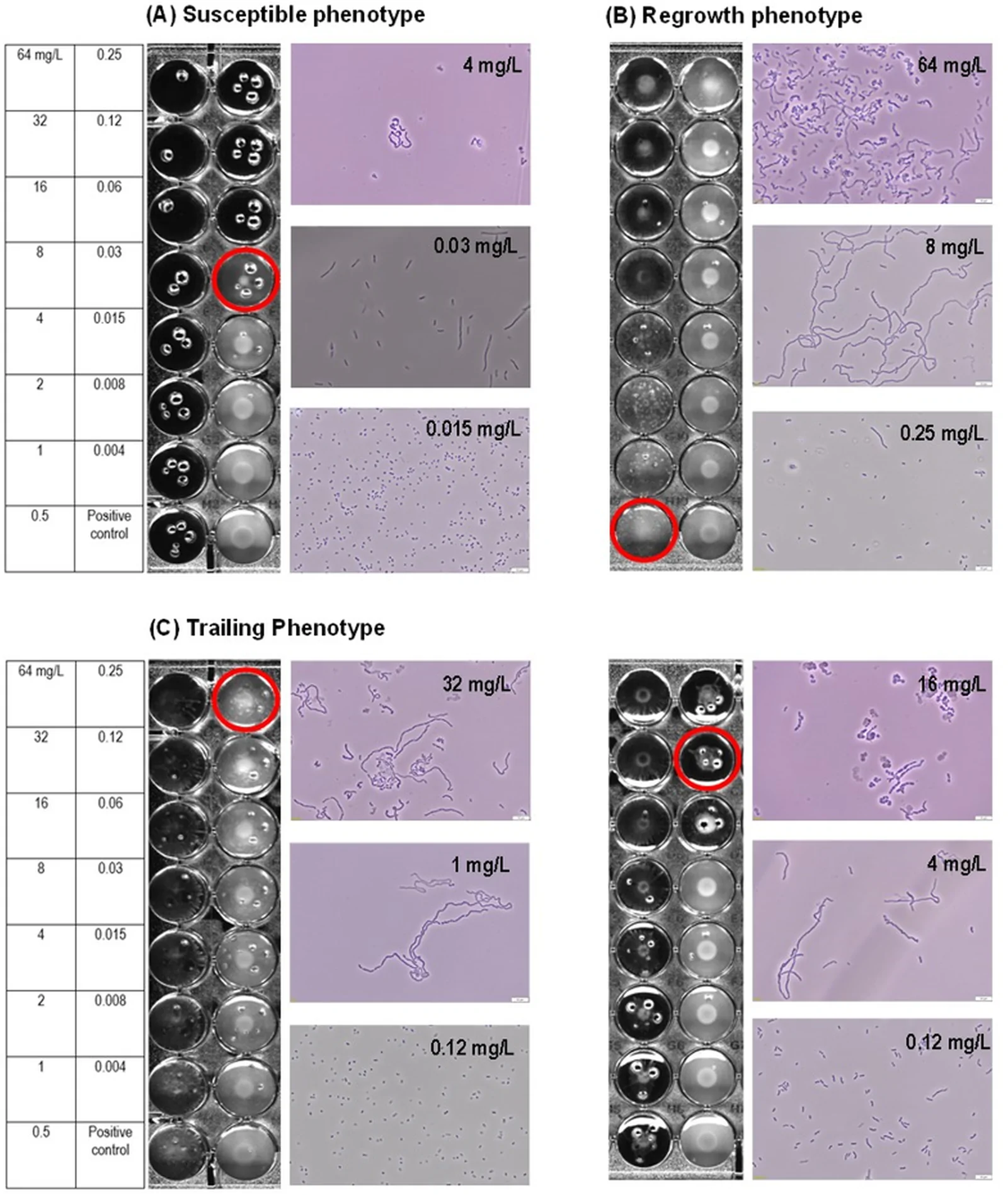
Microscopy images from broth microdilution wells when testing cefiderocol against *Acinetobacter* spp. isolates exhibiting (**A**) susceptibility, (**B**) regrowth, and (**C**) trailing phenotypes (two examples). Cefiderocol MIC values are circled in red, and the concentrations tested are displayed on the left.

A microscopic evaluation of the cellular morphology changes in *Acinetobacter* spp. with different growth patterns in the presence of cefiderocol was also performed. Isolates with clear MIC endpoints had normal coccobacilli cells in the positive control well and in wells below the MIC. A very limited number of cells were noted in the wells above the MIC value (Fig. 4A). Isolates displaying regrowth or trailing phenotypes also exhibited normal coccobacilli cells in the positive control well and in wells below the MIC but presented long filamentous cells reaching up to 195 µm in length at and above the MIC (Fig. 4B and C) or spheroplasts in higher antimicrobial concentrations. The frequency of filamentous cells and spheroplasts appeared to increase as the concentration of cefiderocol in the wells increased (Fig. 4B and C).

### Occurrence of aberrant growth patterns in a surveillance data set

The occurrence of each growth pattern was determined with a subset of *Acinetobacter* spp. clinical isolates (*n* = 455) collected during the 2022 surveillance program evaluating cefiderocol susceptibility in clinical isolates from European and US hospitals. A total of 394 isolates belonging to the *A. baumannii-calcoaceticus* species complex and 61 isolates belonging to 13 other *Acinetobacter* species were included. Isolates were recovered in 13 countries from bloodstream infections (*n* = 48), pneumonia in hospitalized patients (*n* = 162), skin/soft tissue infections (*n* = 49), urinary tract infections (*n* = 14), and unknown or other sites (*n* = 182). Forty percent (182/455) of the *Acinetobacter* spp. clinical isolates displayed a clear MIC endpoint. Within the larger set of surveillance isolates showing no clear endpoints, more granularity to the initial distinct growth patterns, defined as hazy growth and regrowth in the smaller set of *Acinetobacter* isolates initially examined, was observed. A total of 99 (21.8%) isolates displayed hazy growth, including 25 (5.5%) hazy and 74 (16.3%) hazy endpoint (i.e., turbidity with a distinct button of cells in all wells except those at and leading up to the MIC endpoint; Fig. 1B), 120 (26.4%) displayed trailing that consisted of 51 (11.2%) trailing and 69 (15.2%) trailing all the way out (i.e., growth noted above in the highest concentrations tested; Fig. 1E), and 54 (11.9%) exhibited regrowth.

This collection of 455 *Acinetobacter* spp. isolates has a similar cefiderocol MIC distribution as the overall surveillance data set (*n* = 1,042) for 2022 (Fig. S1A), and isolates displaying unclear endpoint growth patterns (hazy, hazy endpoint, regrowth, trail all the way out, and trailing) were equally distributed among isolates with a cefiderocol MIC value >1 mg/L (58%) and ≤1 mg/L (60%; *P* = 0.4464, odds ratio 0.9163 [0.4992-1.6817]). A slightly higher percentage (28% vs 20%) of isolates displaying unclear endpoints were noted at 0.25 mg/L, and the opposite was noted at 0.12 mg/L, where 28% of the isolates showed a clear endpoint and 20% unclear endpoints.

In each country, different growth types were observed, except for Ireland (*n* = 8), Romania (*n* = 1), and Slovenia (*n* = 1), which only showed one growth pattern (Fig. S1B). Notably, Turkey had a high occurrence of isolates displaying trailing, while isolates from Israel displayed hazy phenotypes.

Whole-genome sequencing data were available for 271 of the 455 isolates, and these were examined for molecular features potentially associated with specific growth phenotypes. Core-genome multilocus sequence typing (cgMLST) was used to define the phylogenetic relatedness of these 271 isolates, and multilocus sequence typing identified 34 sequence types (STs) or variants. Notably, excluding singletons, no phylogenetic cluster nor ST was uniquely associated with a single MIC endpoint phenotype (Fig. S2). The presence of acquired class D carbapenemases (oxacillinases) and other β-lactamases/carbapenemases could not be linked to any specific endpoint growth pattern nor could the presence of any alteration within the primary target of cefiderocol in *Acinetobacter*, PBP3 (19). Furthermore, even among isolates clustered closely within the cgMLST phylogeny displaying identical molecular features (i.e., acquired genes and PBP3 genotypes) isolated in the same country or hospital, multiple endpoint growth phenotypes were observed (data not shown).

## DISCUSSION

The presence of trailing and other unclear MIC endpoint patterns when testing *Acinetobacter* spp. isolates against β-lactam agents has been documented in the literature (8, 9). Despite being a known phenomenon, the cause of these unusual patterns is poorly understood. Cefiderocol, like other β-lactams, displays this type of growth behavior during susceptibility testing with *Acinetobacter* spp., and in this study, we aimed to understand the mechanism(s) behind these growth patterns.

Examination of cells in wells displaying turbidity above the MIC demonstrated that those cells were able to escape cefiderocol’s bactericidal activity, despite being damaged metabolically, and revealed impaired cell division with the formation of filamentous and spherical cells. These observations were noted across the different growth patterns that could be distinguished with cefiderocol susceptibility testing (trailing, hazy growth, and regrowth). These findings are reminiscent of the studies by Cross et al. when they evaluated the tolerance mechanisms to meropenem against several Enterobacterales species and demonstrated that the formation of spheroplasts with deficient cell walls allowed for survival of the cells (20). These authors also observed that cells reverted to their normal morphology when meropenem was removed and postulated that this phenomenon allows cells to survive the exposure to bactericidal agents. The same authors subsequently demonstrated that autolysis becomes unbalanced when exposing *Vibrio cholerae* isolates to penicillin and that the cells cannot divide but remain alive (21).

Kriz et al. also reported on the ability of *A. baumannii* and other gram-negative organisms to survive high concentrations of cefiderocol when they noted during mutant selection experiments through serial passage that several isolates tolerated high concentrations of cefiderocol, but once retested, the isolates remained susceptible (22). While the mechanism of this phenomenon was not studied, it was suggested that it could be the result of tolerance related to the same phenomena we observed during susceptibility testing. Additionally, while the observation that many *Acinetobacter* spp. isolates exhibited MBC/MIC ratios greater than 4 *in vitro* may suggest reduced bactericidal activity or tolerance, it is important to recognize that *in vitro* pharmacodynamic parameters do not always directly predict clinical outcomes. Several studies have demonstrated that cefiderocol retains robust efficacy against multidrug-resistant gram-negative pathogens, including carbapenem-resistant *A. baumannii*, in both animal models and clinical settings (23–28).

Our evaluations distinguished five different growth patterns, three of them with unclear MIC endpoints (Fig. 1). The patterns with unclear MIC endpoints appear to be prevalent among clinical isolates, as approximately 60% of the 455 isolates collected in Europe and the United States during a surveillance study displayed those growth types. Isolates displaying unclear MIC endpoints were collected from multiple infection sources, were observed in various countries, and were unrelated to any particular *Acinetobacter* species, genetic profile, or MIC value, but the growth patterns are reproducible. Interestingly, the unclear MIC endpoints did not correlate with the presence of inner colonies, fuzzy edges, or ghost zones noted in the disk diffusion zones of inhibition. The disk diffusion and broth microdilution methods could be used as complementary approaches in cases of uncertainty with isolates showing these unusual endpoint patterns that have MIC or disk zones near the cefiderocol breakpoints. A hallmark of the *Acinetobacter* genus is the extensively clonal nature of circulating isolates down to the country or even hospital level, and this was evident in our phylogenomic analysis. But even among closely clustered isolates with identical molecular profiles, numerous growth phenotypes were observed. For example, 12 ST3 isolates contributed by one hospital in Israel were all cefiderocol susceptible (per CLSI criteria), possessed the same intrinsic *ampC* (ADC-263) and OXA-51 carbapenemase (OXA-71) variants (data not shown), and bore identical G523V alterations in PBP3, and all but one isolate possessed an acquired OXA-23 carbapenemase-encoding gene; 4 of these 12 isolates presented a clear MIC endpoint, while the other 8 did not. While no genetic markers could be identified to explain the growth types at this point, future, more powerful genomic tools that are able to analyze additional and multiple genes may be able to identify such a common mechanism. Alternatively, the growth patterns could be due to differential expression of genes controlling peptidoglycan biosynthesis, either through mutation or the intragenomic movements of transposable elements. Further phenotypic differentiation (i.e., trailing vs haziness) could be driven by strain-level differences stemming from unique mutations or plasmids in individual isolates. Transcriptome analysis of closely related isolates with unique growth patterns in the presence of different cefiderocol concentrations (below, at, and above the MIC) could help elucidate the molecular mechanism(s) of the observed growth patterns with this genus when exposed to cefiderocol.

Both CLSI and EUCAST organizations provide guidance for reading cefiderocol tested in the reference broth microdilution, and our study does not propose modifications in the reading recommendations to facilitate endpoint determination when testing cefiderocol. Despite these recommendations, trailing, hazy growth, and regrowth generate uncertainty when calling MIC endpoints, and determining the MIC becomes subjective and challenging for inexperienced readers. The clinical impact of the growth patterns remains unclear. However, studies have identified environmental parameters (pH and/or the presence of bicarbonate) that can reduce trailing, so the effect is possibly an *in vitro* testing artifact (29). Because of its good *in vitro* and *in vivo* activity (19) and due to the limited therapeutic options available to treat infections caused by *Acinetobacter* spp. isolates that are often MDR, testing and reporting cefiderocol against these organisms could benefit patients. In summary, trailing, hazy growth, and regrowth when testing cefiderocol against *Acinetobacter* spp. by the broth microdilution methodology are reproducible and appear to reflect β-lactam activity without killing the cells that are morphologically damaged. Understanding this phenomenon can assist in understanding the CLSI reading guidance and in determining when to perform repeat disk diffusion testing or use another method to confirm the result when MIC values are close to the breakpoint for this agent.

## MATERIALS AND METHODS

### Bacterial isolates

All clinical isolates described in this study were collected as part of the SENTRY Antimicrobial Surveillance Program. Briefly, isolates recovered from bloodstream, intra-abdominal, urinary tract, skin, and skin structure infections and from patients hospitalized with pneumonia were submitted to a central testing laboratory (Element Iowa City/JMI Laboratories, North Liberty, IA) by hospitals located in Asia-Pacific, Europe, Latin America, and North America. These isolates were considered the cause of infections by local criteria, and only one isolate per patient episode was submitted to the study. Bacterial identification was performed in local laboratories and confirmed as needed in the central laboratory by MALDI-TOF MS.

*Acinetobacter* spp. isolates (*n* = 23) were selected from the 2020 surveillance year based on phenotypes observed during initial BMD testing and included isolates exhibiting trailing (*n* = 5), haziness (*n* = 5), regrowth (*n* = 5), resistance with robust growth up to the highest antimicrobial concentration tested (*n* = 4), and clear in-range susceptible result (*n* = 4). The characteristics of these isolates are summarized in Table 1. For the purposes of this study, the endpoint growth phenotypes were defined as follows: trailing, reduced but persistent growth across consecutive concentrations above the first well showing marked growth reduction; hazy growth, a homogenous, faintly turbid suspension without a discrete growth button, present in wells above the apparent endpoint; and regrowth, distinct growth reappearing at concentrations above one or more wells that showed inhibition, reminiscent of the Eagle effect. Resistant isolates showed robust growth up to the highest concentration tested, and susceptible isolates showed a clear, abrupt growth endpoint within the tested range. Endpoints were read by a single experienced reader applying these definitions and the current CLSI reading guidance. Additionally, 455 *Acinetobacter* spp. isolates tested consecutively during 4 months of the 2022 SENTRY Program were selected from the 1,042 isolates included in the surveillance program and evaluated to determine the prevalence of the growth phenotypes described in this study.

### Antimicrobial susceptibility testing methods

The reference broth microdilution susceptibility testing method was performed following the CLSI guidelines (30). Cefiderocol was tested in IDCAMHB prepared by Chelex treatment methods detailed in the CLSI guidelines (31). Two lots of iron-depleted test media were generated in-house from a single lot of MHB powder (cat. no. 212322; Becton, Dickinson and Company; New Jersey, USA). Iron depletion was confirmed using the Visocolor HE Iron kit (Macherey-Nagel, Düren, Germany) following the manufacturer’s instructions. Cefiderocol patient vials supplied by Shionogi, Inc. were used in all experiments. MIC results were interpreted using the current CLSI cefiderocol breakpoints for *Acinetobacter* spp. (susceptible, ≤4 mg/L; intermediate, 8 mg/L; and resistant, ≥16 mg/L), and isolates were categorized as susceptible or resistant accordingly (31). For the 23 selected isolates, reproducibility was assessed by retesting each isolate in five replicates, with each replicate prepared from an independent inoculum on a separate day using the same lot of IDCAMHB and BMD panels. Endpoints for both the initial and the replicate testing were determined by the same single experienced reader to minimize inter-reader variability. Isolates were maintained as frozen stocks at −80°C between the initial surveillance testing and the replicate, disk diffusion, MBC, viability, and microscopy experiments described below, and were subcultured no more than twice before testing.

Disk diffusion testing followed CLSI guidelines (32). Isolates were tested in triplicate using separate inocula, commercially available MHA (BD BBL) media, and cefiderocol disks (30 μg, Hardy Diagnostics). Growth observations, including the presence of colonies within the zone of inhibition, were documented alongside the measured zone diameter. Discrete colonies or rare small inner colonies were ignored when measuring zone diameter.

Quality control for antimicrobial susceptibility testing methods was performed by concomitantly testing *Escherichia coli* ATCC 25922, *Pseudomonas aeruginosa* ATCC 27853, and *P. aeruginosa* SR27001, the last of which displays higher MIC values in the presence of iron.

### Minimum bactericidal concentration testing

MBC testing was performed by resuspending, plating, and counting the colony-forming units of the cell suspension used to inoculate MIC panels and the growth in the MIC wells after overnight incubation (33). The MBC was determined at the concentration of antimicrobial agent that resulted in a 1,000-fold reduction of viable bacterial colonies after incubation compared to the initial inoculum (34). An *in vitro* definition of bactericidal activity was applied using the ratio of MBC/MIC of ≤4 (35).

### Determination of cell viability in broth microdilution wells

*Acinetobacter* spp. cell viability in BMD panels containing cefiderocol was assessed using an oxidase reduction indicator, resazurin, that produces a blue to pink color change in the presence of metabolically active cells. The resazurin-based reagent alamarBlue was applied following the manufacturer’s instructions. Once cefiderocol susceptibility testing was completed and endpoints were determined, 10 µL of alamarBlue HS Cell Viability Reagent (cat. no. A50101; Invitrogen; Waltham, MA) was added to each well and incubated for 1 hour at 37°C in ambient atmosphere. Additionally, alamarBlue was added to an uninoculated negative control panel containing only IDCAMHB. Color change observations and OD (570 nm) reads were recorded with particular attention to wells surrounding the MIC endpoint.

### Microscopic evaluation of trailing well contents

A subset of eight isolates with different growth patterns were selected for microscopic evaluation (two regrowth, two hazy, two resistant, and two susceptible). Growth from susceptibility test wells below, at, and above the MIC for isolates of each phenotype was sampled and observed under an Olympus MX53 microscope (Olympus Corporation, Tokyo, Japan) at 100× magnification. Cell morphology observations were recorded; photos were taken using an Olympus DP23 digital microscope camera; and images were analyzed using the Olympus cellSens software v3.

### Molecular characterization of *Acinetobacter* spp. isolates

Two hundred seventy-one isolates with available genome sequencing data were characterized for mechanisms potentially affecting endpoint growth phenotypes. These isolates were randomly selected from the 455 isolate surveillance subset, and whole-genome sequencing was performed on total genomic DNA using a MiSeq or NextSeq platform according to the manufacturer’s instructions. A phylogenetic tree of these isolates was constructed based on the cgMLST of *A. baumannii* containing 2,390 genes (36) available at cgMLST.org using chewBBACA v3.2.0 (37). A multiple genome alignment was performed using MUSCLE v3.8.11 (38), and an unrooted maximum likelihood tree was created with FastTree v2.2.11 using default settings (39). The maximum likelihood tree and associated metadata were curated with iTol v6.8.2 (40).

To assess isolates for the presence of acquired genes and alterations in PBP3 and to determine Pasteur scheme MLST, available FASTQ format files for each sample set were assembled independently using the *de novo* assembler SPAdes, using the as-then current version, with *K* values of 21, 33, 55, 77, and 99 plus “careful mode” on to reduce the number of mismatches (41). An in-house proprietary bioinformatics pipeline and a curated resistance gene database based on the National Center for Biotechnology Information Bacterial Antimicrobial Resistance Reference Gene Database (https://www.ncbi.nlm.nih.gov/bioproject/PRJNA313047) were used for the *in silico* screening of β-lactamase genes. All genes were used as queries to align against the target assembled sequences. MLST was determined by extracting the relevant alleles from the sequencing data for each isolate and querying against the database available at pubmlst.org. When no type was available (novel types, single or multilocus variants), “~” was appended to the nearest available ST. Alterations in PBP3 were determined relative to *A. baumannii* ATCC 19606.

## Data Availability

Whole-genome sequencing data for the isolates analyzed in this study have been deposited in the NCBI Sequence Read Archive under BioProject accession number PRJNA1481234.

## SUPPLEMENTAL MATERIAL

Fig. S1 jcm.00186-26-s0001.tif (A) Distribution of Acinetobacter spp. isolates from 2022 surveillance, the subset analyzed for MIC endpoint phenotypes, isolates displaying clear and unclear (hazy, regrowth, and trailing) endpoints; (B) Distribution of clear, hazy, regrowth, and trailing endpoints by country.

Fig. S2 jcm.00186-26-s0002.tiff Genetic analysis of surveillance isolates.

Table S1 jcm.00186-26-s0003.docx Molecular characteristics of 271 *Acinetobacter* spp. clinical isolates from surveillance sequenced and stratified by growth phenotype.

ASM does not own the copyrights to Supplemental Material that may be linked to, or accessed through, an article. The authors have granted ASM a non-exclusive, world-wide license to publish the Supplemental Material files. Please contact the corresponding author directly for reuse.
