# Supplementary material for: Evaluation of cefiderocol trailing and other unclear susceptibility testing endpoints for Acinetobacter spp
Source: J Clin Microbiol. 2026 Aug 20;64(9):e00186-26. doi: 10.1128/jcm.00186-26 (PMC13556250; doi:10.1128/jcm.00186-26)
Supplement: Supplementary file 1 [file jcm.00186-26-s0003.docx]

**Supplemental Table 1** Molecular characteristics of 271 *Acinetobacter* spp. clinical isolates from surveillance sequenced and stratified by growth phenotype

| **Molecular Attribute^a^** | **Growth phenotype (no. of isolates)/No. of isolates (%)** | | | | | | |
| --- | --- | --- | --- | --- | --- | --- | --- |
|  | **Clear (102)** | **Hazy (13)** | **Hazy Endpoint**  **(62)** | **Regrowth (19)** | **Trail-AWO^b^**  **(53)** | **Trailing (22)** | **Total**  **(271)** |
| Sequence Type (ST) |  |  |  |  |  |  |  |
| 2 | 78 (40.4) | 9 (4.7) | 42 (21.8) | 11 (5.7) | 39 (20.2) | 14 (7.3) | 193 (100.0) |
| 3 | 10 (35.7) | 0 (0) | 5 (17.9) | 3 (10.7) | 6 (21.4) | 4 (14.3) | 28 (100.0) |
| 78 | 5 (62.5) | 0 (0) | 2 (25.0) | 1 (12.5) | 0 (0) | 0 (0) | 8 (100.0) |
| 499 | 2 (33.3) | 0 (0) | 2 (33.3) | 0 (0) | 1 (16.7) | 1 (16.7) | 6 (100.0) |
| 150 | 1 (25.0) | 0 (0) | 1 (25.0) | 1 (25.0) | 1 (25.0) | 0 (0) | 4 (100.0) |
| 570 | 1 (33.3) | 0 (0) | 1 (33.3) | 1 (33.3) | 0 (0) | 0 (0) | 3 (100.0) |
| 15 | 1 (50.0) | 0 (0) | 0 (0) | 0 (0) | 1 (50.0) | 0 (0) | 2 (100.0) |
| Other | 4 (14.8) | 4 (14.8) | 9 (33.3) | 2 (7.4) | 5 (18.5) | 3 (11.1) | 27 (100.0) |
| PBP3 Genotype |  |  |  |  |  |  |  |
| A515V | 59 (39.9) | 6 (4.1) | 27 (18.2) | 11 (7.4) | 34 (23) | 11 (7.4) | 148 (100.0) |
| A583V | 1 (50.0) | 0 (0) | 0 (0) | 0 (0) | 1 (50.0) | 0 (0) | 2 (100.0) |
| D56N, T106A, L177I | 1 (100.0) | 0 (0) | 0 (0) | 0 (0) | 0 (0) | 0 (0) | 1 (100.0) |
| F48L | 0 (0) | 0 (0) | 0 (0) | 1 (100.0) | 0 (0) | 0 (0) | 1 (100.0) |
| G523V | 10 (35.7) | 0 (0) | 5 (17.9) | 3 (10.7) | 6 (21.4) | 4 (14.3) | 28 (100.0) |
| H370Y | 0 (0) | 0 (0) | 1 (100.0) | 0 (0) | 0 (0) | 0 (0) | 1 (100.0) |
| I53F | 0 (0) | 0 (0) | 1 (100.0) | 0 (0) | 0 (0) | 0 (0) | 1 (100.0) |
| K235N | 0 (0) | 0 (0) | 1 (100.0) | 0 (0) | 0 (0) | 0 (0) | 1 (100.0) |
| N392T | 4 (80.0) | 0 (0) | 0 (0) | 0 (0) | 1 (20.0) | 0 (0) | 5 (100.0) |
| Q488H | 0 (0) | 0 (0) | 0 (0) | 1 (100.0) | 0 (0) | 0 (0) | 1 (100.0) |
| T511S | 0 (0) | 0 (0) | 0 (0) | 0 (0) | 0 (0) | 1 (100.0) | 1 (100.0) |
| T526S | 3 (100.0) | 0 (0) | 0 (0) | 0 (0) | 0 (0) | 0 (0) | 3 (100.0) |
| V581I, V595I | 0 (0) | 0 (0) | 0 (0) | 0 (0) | 0 (0) | 1 (100.0) | 1 (100.0) |
| ND | 2 (66.7) | 0 (0) | 1 (33.3) | 0 (0) | 0 (0) | 0 (0) | 3 (100.0) |
| WT | 22 (29.7) | 7 (9.5) | 26 (35.1) | 3 (4.1) | 11 (14.9) | 5 (6.8) | 74 (100.0) |
| OXA-23-like presence |  |  |  |  |  |  |  |
| OXA-23-like | 79 (40.3) | 6 (3.1) | 37 (18.9) | 14 (7.1) | 44 (22.4) | 16 (8.2) | 196 (100.0) |
| ND | 22 (29.7) | 7 (9.5) | 25 (33.8) | 5 (6.8) | 9 (12.2) | 6 (8.1) | 74 (100.0) |
| OXA-24-like presence |  |  |  |  |  |  |  |
| OXA-24 | 0 (0) | 0 (0) | 3 (75.0) | 0 (0) | 1 (25.0) | 0 (0) | 4 (100.0) |
| OXA-72 | 19 (41.3) | 3 (6.5) | 17 (37.0) | 2 (4.3) | 3 (6.5) | 2 (4.3) | 46 (100.0) |
| ND | 83 (37.6) | 10 (4.5) | 42 (19.0) | 17 (7.7) | 49 (22.2) | 20 (9.0) | 221 (100.0) |

a ND= not detected; WT= wildtype

b AWO= all the way out

**Supplemental Figure 1** (A) Distribution of *Acinetobacter* spp. isolates from 2022 surveillance, the subset analyzed for MIC endpoint phenotypes, isolates displaying clear and unclear (hazy, regrowth and trailing) endpoints; (B) Distribution of clear, hazy, regrowth and trailing endpoints by country

**Supplemental Figure 2** Phylogenetic reconstruction of 271 *Acinetobacter baumannii-calcoaceticus* complex isolates. Colored rings indicate isolate data associated with (from innermost to outermost): endpoint growth phenotype (clear, green; hazy endpoint, orange; trailing, blue; regrowth, teal; trailing all the way out, yellow; hazy, red), country (regional coloration for USA, red; Western Europe, green; Eastern Europe, purple; Mediterranean, brown), multi-locus sequence type (Pasteur scheme: ST2, dark blue; ST3, grey; ST78, purple; ST499, yellow; ST represented by ≤4 isolates light blue, see Supplemental Data for corresponding STs), class D OXA-type carbapenemase (OXA-23-like, red; OXA-24, light blue; OXA-72, dark blue; OXA-23-like+OXA-72, purple; none detected, open), other β-lactamase (TEM-1, light blue; NDM-1+TEM-1, red; CARB-16, blue; CTX-M-115, dark blue; none detected, open), and PBP3 alteration (wild-type, open; A515V, yellow; G523V, red; other, blue, see Supplemental Data for specific alterations; not determined, open).
